# Evidence-based unification of potato gene models with the UniTato collaborative genome browser

**DOI:** 10.3389/fpls.2024.1352253

**Published:** 2024-06-11

**Authors:** Maja Zagorščak, Jan Zrimec, Carissa Bleker, Nadja Nolte, Mojca Juteršek, Živa Ramšak, Kristina Gruden, Marko Petek

**Affiliations:** Department of Biotechnology and Systems Biology, National Institute of Biology, Ljubljana, Slovenia

**Keywords:** *Solanum tuberosum*, bioinformatics analysis, plant genome annotation, Solanaceae, gene model annotations, Phureja group, GFF files

## Abstract

Potato (*Solanum tuberosum*) is the most popular tuber crop and a model organism. A variety of gene models for potato exist, and despite frequent updates, they are not unified. This hinders the comparison of gene models across versions, limits the ability to reuse experimental data without significant re-analysis, and leads to missing or wrongly annotated genes. Here, we unify the recent potato double monoploid v4 and v6 gene models by developing an automated merging protocol, resulting in a Unified poTato genome model (UniTato). We subsequently established an Apollo genome browser (unitato.nib.si) that enables public access to UniTato and further community-based curation. We demonstrate how the UniTato resource can help resolve problems with missing or misplaced genes and can be used to update or consolidate a wider set of gene models or genome information. The automated protocol, genome annotation files, and a comprehensive translation table are provided at github.com/NIB-SI/unitato.

## 1 Introduction

*Solanum tuberosum* (potato) is among the most important food crops and a model tuber species. The crop is highly useful as an organism for studying plant responses to environmental stress factors (Lukan et al., 2022; Bleker et al., 2024), such as herbivory (Petek et al., 2020a), viral diseases (Baebler et al., 2020), transcriptional (Tomaž et al., 2023) and small RNA regulation (Križnik et al., 2020), single and combined abiotic stress responses (Demirel et al., 2020), and growth-defense trade-offs (Huot et al., 2014). Potato also serves as an excellent platform for transferring and testing vast amounts of knowledge garnered in *Arabidopsis* with an agriculturally relevant crop (Ramšak et al., 2018; Zagorščak et al., 2018; Schwacke et al., 2019), aiding toward solving present-day food security issues (Cole et al., 2018).

Apart from novel wild potato (Tang et al., 2022) and pan-genome assemblies (Hoopes et al., 2022; Bozan et al., 2023), the double monoploid (DM) clone of group Phureja DM1–3 516 R44 was until recently the standard variety on which gene models were defined. DM genome assemblies and gene models have been introduced by multiple consortia, including the Potato Genome Sequencing Consortium (PGSC), International Tomato Genome Consortium (ITAG), and Buell Lab (University of Georgia). These sequenced and assembled (Yandell and Ence, 2012) up to 88% of the potato genome that included between 35,004 (ITAG) (Tomato Genome Consortium, 2012) and 39,428 (PGSCv4.04) (Potato Genome Sequencing Consortium et al., 2011) gene models, with the recent nanopore long read assembly DMv6.1 (Pham et al., 2020) annotating 40,652 genes (in the working version). Moreover, we previously unified the PGSC v4 and ITAG gene models into a merged DMv4n version containing 49,322 genes (Petek et al., 2020b).

In contrast to *Arabidopsis* gene models, where version control and gene model tracking have been utilized for over a decade and gene annotations are optimized (Rhee et al., 2003; Lamesch et al., 2012), this is not the case with potato and similar less matured crop assemblies. Here, sequencing assemblies and gene models are only slowly advancing, and while each subsequent version improves sequencing depth, coverage, and assembly statistics, the gene models are reformulated. Frequently, previous gene model versions are not accounted for, and mapping and translation tables are not provided (e.g., Pham et al., 2020; Hoopes et al., 2022). This unfortunately limits potato research in multiple ways: i) hindering comparison of gene models across versions and experiments, ii) limiting the reuse and integration of experimental data based on older model versions (e.g., v4) with the latest version (v6) without extensive reprocessing of the RNA-Seq data, and iii) impeding the use of certain popular comparative genomics resources, such as Plaza and Ensembl plants (Valentin et al., 2021; Van Bel et al., 2022; Yates et al., 2022), which, as of writing this paper, have not been updated to the latest gene model versions (v6). Plaza is a platform for comparative, evolutionary, and functional plant genomics, which even in its latest version (5.0) uses v4 potato gene models (Van Bel et al., 2022). On the other hand, Ensembl plants (Yates et al., 2022), a plant genome analysis platform, is based on even older PGSC v3 potato gene models (Visser et al., 2009), yet is a source for other derived ontology (Schwacke et al., 2019) and transcription factor databases (Tian et al., 2020).

In addition to the previously mentioned issues, inadequate consideration of previous gene model information has resulted in the omission of a number of known genes (Visser et al., 2009; Potato Genome Sequencing Consortium et al., 2011; Tomato Genome Consortium, 2012; Petek et al., 2020b). In the case of the recent v6 gene models, we observed that they do not include certain well-known genes with important molecular functions, as they do not account for previous gene model information (Pham et al., 2020). An example is the transcription factor TGA2, an essential regulator of hormonal signaling (Tomaž et al., 2023). Aside from such missing genes, some are also moved, merged, or split (as presented in the sections below). These deviations can lead to differences in interpretation in downstream analyses (e.g., gene family expansion, differential expression, marker selection, gene set enrichment analysis) (Yandell and Ence, 2012). In addition to these imperfect annotations negatively affecting future experiments, existing published results using previous gene models, including, e.g., AlphaFold structure predictions (Tomaž et al., 2023), have become outdated, making it essential to update and consolidate gene predictions.

To help resolve these issues, here, we expand the ITAG and PGSC v4 annotations with v6 annotations (Pham et al., 2020), unifying the different gene models. In addition, we include experiment-based evidence from our pan-transcriptome (Petek et al., 2020b), short- and long-read sequencing data (Lukan et al., 2020; Hoopes et al., 2022), and Solanaceae proteomes (Hosmani et al., 2019; Wang et al., 2024), thereby creating an improved and more accurate potato gene annotation model for downstream analyses. To ensure the transparency and accuracy of future gene models, we present the Unified poTato genome annotation resource (UniTato). UniTato is provided through an Apollo web interface (Dunn et al., 2019), enabling a community-driven effort for real-time revision and enhancement of gene models by experts. This will increase the interpretational power of experimental datasets and facilitate the reuse of experimental analyses conducted on v4, thus expediting progress in potato research.

## 2 Results

### 2.1 A unified v4 and v6 potato genome annotation

**Figure 1:**
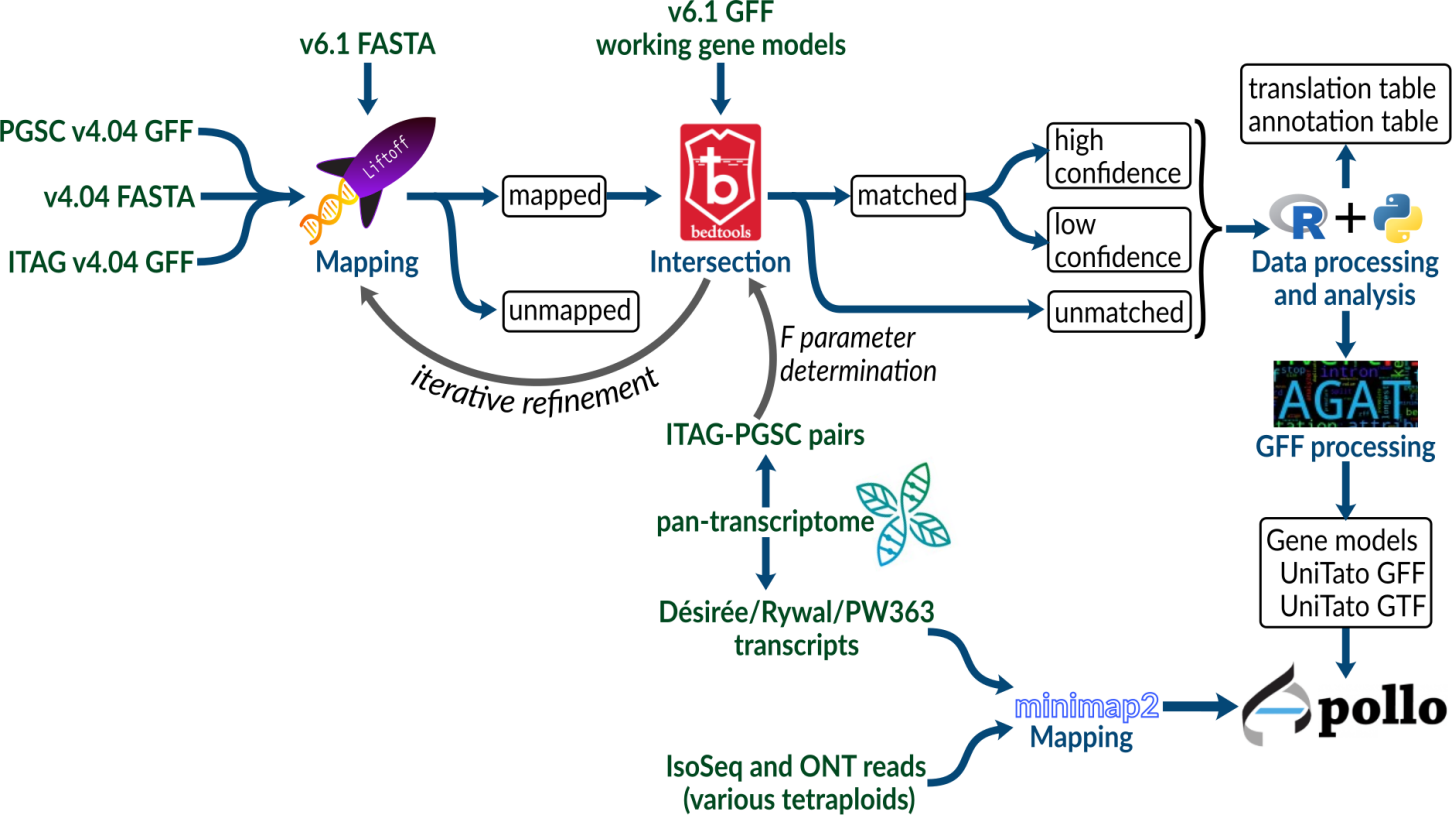
Schematic overview of the procedure used to create a unified DM v4 and v6 potato genome annotation resource.

To compare the potato gene model versions, we mapped gene annotations of older PGSCv4.04 (Potato Genome Sequencing Consortium et al., 2011) and ITAG assemblies (Tomato Genome Consortium, 2012) to the recent potato DMv6.1 assembly (Pham et al., 2020) using Liftoff (Shumate and Salzberg, 2020) and used Bedtools *intersect* (Quinlan and Hall, 2010) to find intersecting genes (**Figure 1**, see Methods 2.2). Briefly, Liftoff is a tool that accurately maps annotations between assemblies of the same or closely related species. We used it to transfer the gene model annotations from v4 to the v6 assembly. Two genome assemblies (either ITAG or PGSC v4 and DMv6.1) and a v4 annotation file (ITAG or PGSC v4, respectively) were provided as input. The v4 gene models were aligned chromosome by chromosome to the v6 genome assembly. Bedtools *intersect* (Quinlan and Hall, 2010) was then used to check for overlap (intersection) between the sets of v4 and v6 gene models.

We first explored the Liftoff *flank* parameter, which controls the amount of flanking sequence upstream and downstream of a gene, by using a setting of either none or 500 nt. In order to include the gene neighborhood, the upstream and downstream expansion of each v4 gene sequence (combined PGSC/ITAG v4 dataset) before mapping can improve mapping precision. This is especially important for the ITAG annotation which contains only CDS regions, as opposed to the PGSC annotation, where complete mRNA sequences are provided. Without a flanking sequence (0 nt), we mapped 72,143 v4 gene models, whereas when using a flanking sequence of 500 nt length, we mapped 73,820 v4 gene models. Using either of the *flank* parameter settings, 316 PGSC and 211 ITAG gene models could not be mapped to the v6 genome assembly (**Table 1**; **Supplementary Table S1**).

**Table 1:** Overview of total gene counts and Liftoff results at different flank parameter values for v4 and v6 gene models.

	Total gene count	No *flank*, unmapped	*Flank* 500 nt, unmapped	No *flank*, mapped	*Flank* 500 nt, mapped
**PGSC (v4)**	39,428 (100.00%)	492 (1.25%)	363 (0.92%)	38,936 (98.75%)	39,065 (99.08%)
**ITAG (v4)**	35,004 (100.00%)	1,797 (5.13%)	249 (0.71%)	33,207 (94.87%)	34,755 (99.29%)
**DMV6.1 working**	40,652 (32,917 hc)	/	/	/	/

Three hundred sixteen PGSC and 211 ITAG gene models could not be mapped to the v6 genome assembly (unmapped) with either flank parameter value.

hc, gene models defined as “high confidence” in v6 (Pham et al., 2020).

**Figure 2:**
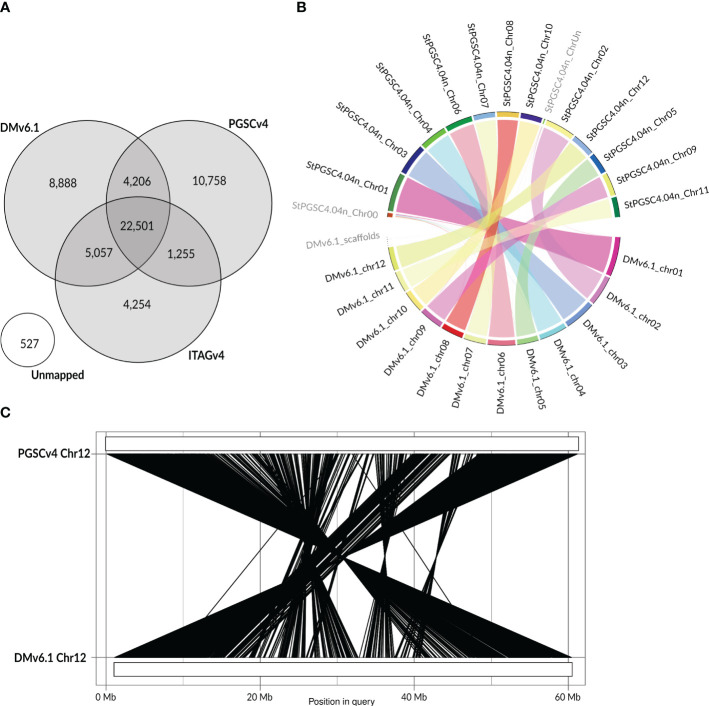
Mapping v4 to v6 gene models. **(A)** Venn diagram of overlaps between the v4 and v6 gene models obtained using Liftoff and Bedtools *intersect* (*F* > 0.30). In the intersected areas, note that the number of v6 IDs is shown. **(B)** Chord diagram of the synteny between v4 and v6 gene models. The diagram shows that most chromosomes are almost completely syntenic across models; however, some scaffolds remain unanchored. **(C)** Rearrangements of chromosome 12 in v6 genome assembly vs. the v4 genome assembly. The lines represent synteny between gene model coding regions. Other chromosomes’ pairwise synteny graphs can be found on the Unitato GitHub page (https://github.com/NIB-SI/unitato).

Next, to identify the overlap between the sets of v4 and v6 gene models, we explored the Bedtools *F* parameter, which allows for control over the minimum overlap required as a fraction of the length of v4 gene models. By ranging *F* from 0.0001 to 1, we found that 0.30 was the optimum value (**Supplementary Figure S1**; **Table 2**). With the Liftoff *flank* parameter of 500 nt, we achieved a mapping coverage (*F* >= 0.3, high identity) with 56,776 v4 gene models mapping to 31,594 v6 models [of these, 92% belong to v6 high confidence gene models as defined by Pham et al. (2020), **Supplementary Table S1**]. Since *flank* can also capture v4 assembly gaps (N runs) or misassemblies that were corrected in the v6 assembly, using it may not always be the optimal choice. For example, we found that no flanking sequence (0 nt) achieved a better mapping coverage *F* with 387 v4 gene models mapping to 458 v6 models. We thus decided to keep the Liftoff result with the better mapping coverage per gene (either 0 or 500 nt *flank*), as reported above. For gene models with a Bedtools coverage *F* above or equal to 0.30, we kept the v6 gene models and added 17,272 v4 models with low coverage (*F* < 0.30). This merge resulted in the final genome annotation model, termed UniTato (**Figures 2A, B**). Note that the v6 genome assembly has many inversions compared to the v4 assembly, most evidently in chromosome 12 (**Figure 2C**).

**Table 2:** Coverage of v4 to v6 gene models by the number of models and % of all v6 models, at different Bedtools *intersect* sequence coverage (*F*) parameter values.

	Version	*F* = 1	*F* >= 0.30	*F* >= 0.0001
**PGSC/ITAG no *flank*, working version**	v4	40,252 (54.08%)	56,512 (75.92%)	57,663 (77.47%)
	v6	27,103 (66.67%)	31,481 (77.44%)	32,263 (79.36%)
**PGSC/ITAG no *flank*, high confidence**	v4	38,095 (51.18%)	53,360 (71.69%)	54,199 (72.81%)
	v6	25,331 (76.95%)	28,986 (88.06%)	29,470 (89.53%)
**PGSC/ITAG *flank* 500 nt, working version**	v4	40,586 (54.53%)	57,040 (76.63%)	58,209 (78.20%)
	v6	27,261 (67.06%)	31,669 (77.90%)	32,452 (79.83%)
**PGSC/ITAG *flank* 500 nt, high confidence**	v4	38,373 (51.55%)	53,793 (72.27%)	54,637 (73.40%)
	v6	25,443 (77.29%)	29,113 (88.44%)	29,586 (89.88%)

Genes that mapped with the same F value with and without flank are counted twice.

Of the observed 17,272 v4 gene models with low coverage (*F* < 0.30), 11,832 were from the PGSC dataset and 5,440 from ITAG. These sequences are present in the v6 assembly but were not identified as genes (Pham et al., 2020). We decided to retain all such “rescued” genes and assigned them with the identifier from v4. Of these, 16,117 mapped to the intergenic regions in v6 (*F* < 0.0001). On the other hand, 8,888 v6 working version gene models were not supported by v4 annotations (of these, 5,979 with v6 annotation “hypothetical protein”), of which 3,742 were high-confidence v6 gene models. Finally, we further analyzed the genome-mapped and unmapped v4 genes, searching for evidence of their expression within our published pan-transcriptome dataset (Petek et al., 2020b). The v4 gene models that do not match any v6 gene models (*F* < 0.0001) but do match tetraploid transcriptomes (3,596 out of 15,590 gene models) were considered to be valid genes. On the other hand, some of the 11,924 gene models that match neither the v6 models nor the pan-transcriptome are likely unreliable gene model predictions. Note that 292 out of 559 v4 gene models did not map to the v6 genome yet match tetraploid Désirée, Rywal, or PW363 transcripts. These genes were lost with the reassembly of the DM scaffold in v6 (Pham et al., 2020). Moreover, we obtained additional evidence about the reliability of the transcriptome-unsupported rescued genes, by mapping to the genome RNA-Seq reads of DM Phureja and tetraploid cultivars (Lukan et al., 2020; Petek et al., 2020b; Hoopes et al., 2022) as well as protein sequences of *Arabidopsis* (Cheng et al., 2017; Pasha et al., 2020) and three *Solanaceae* species (Hosmani et al., 2019; Wang et al., 2024) (see Results ch. 2.2). Finally, the newly generated GFF3 file and a table linking identifiers of ITAG and PGSC v4 gene models with v6 gene models are available at GitHub (https://github.com/NIB-SI/unitato).

### 2.2 UniTato database access and user interface

**Figure 3:**
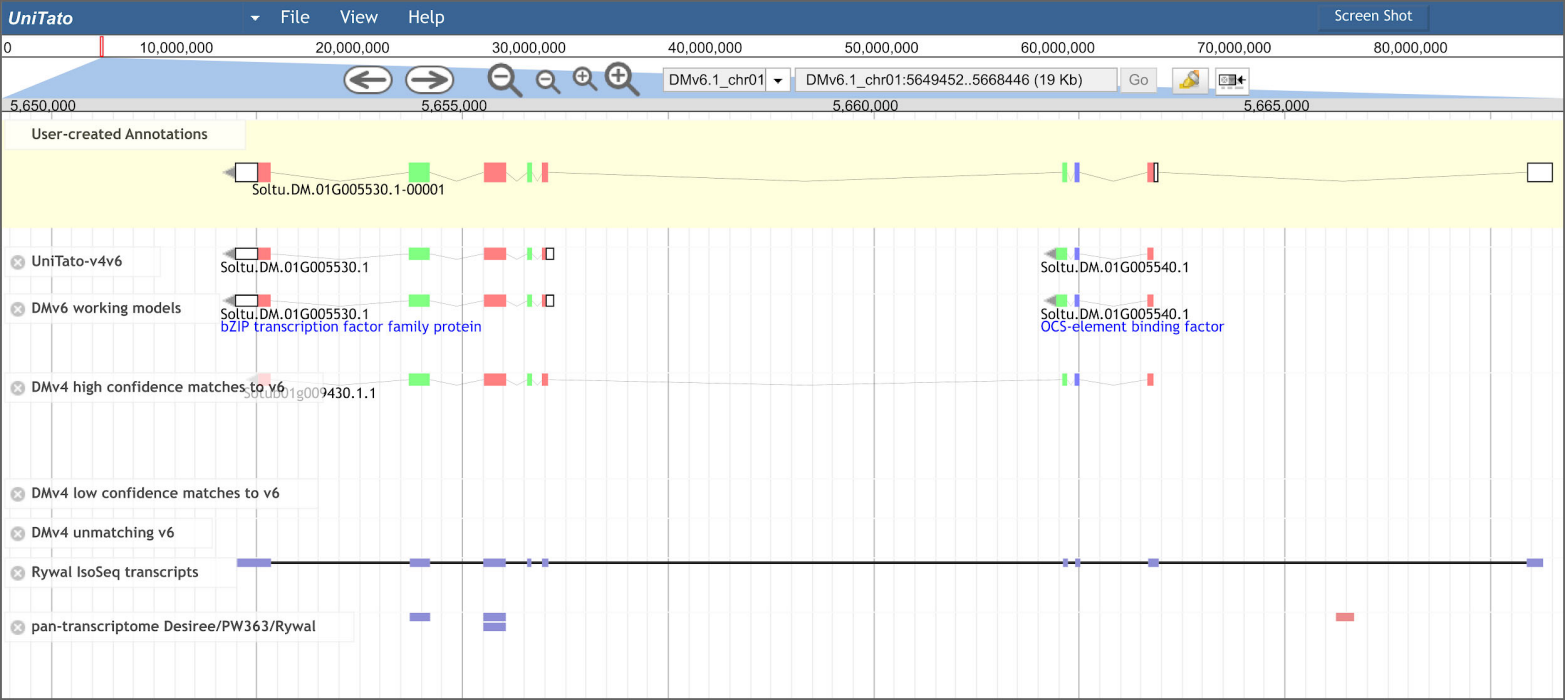
Overview of the UniTato user interface and TGA2 use case. Screenshot of the Apollo server web interface for the *Solanum tuberosum* DM gene model manual annotation, showing the manual annotation of a TGA2 transcription factor gene model which was split into two gene models in v6 (track “DMv6 working models”). The gene model’s manual annotation with nine exons (track “User-created Annotations”) was based on the correctly predicted ITAG v4 CDS and the Rywal Iso-Seq transcript mapping.

The UniTato database (accessible at http://unitato.nib.si/) is hosted in a deployment of the community-focused genome annotation editor Apollo (Dunn et al., 2019) (**Figure 3**). Based on the popular JBrowse genome viewer (Buels et al., 2016), Apollo allows visitors to browse, compare, and interpret the available evidence-based gene models. The annotator panel in the Apollo interface provides several tabs, allowing easy navigation through the genome and the ability to view or hide tracks as well as to locate and view annotation details. For further information, we refer the reader to the Apollo documentation (https://genomearchitect.readthedocs.io/).

The Apollo interface currently contains a number of tracks (see **Table 3**, **Supplementary Table S2**), which include various gene models (v4, v6, unified v4 and v6) as well as different subsets of high-confidence matching and rescued genes. To aid in interpreting and evaluating the gene models, a number of evidence tracks are also available, including long read and short paired-end Illumina mappings from DM Phureja and tetraploid cultivars (Lukan et al., 2020; Petek et al., 2020b; Hoopes et al., 2022), reference proteomes of Arabidopsis (*Arabidopsis thaliana*) (Cheng et al., 2017; Pasha et al., 2020), tomato (*Solanum lycopersicum*) (Hosmani et al., 2019), tobacco (*Nicotiana tabacum*) and *Nicotiana benthamiana* (Wang et al., 2024), and reference transcriptomes of potato cultivars Désirée, PW363, and Rywal (Petek et al., 2020b). These tracks are publicly viewable by all UniTato web page visitors. On the other hand, potential contributors are encouraged to use the contact details on the web page to request edit access through a user account. Upon login, these users have access to the curator tools, providing the ability to collaboratively add, remove, and modify potato gene models. The improvements can then be exported as an updated version of the genome annotation file (GFF, VCF, or FASTA).

**Table 3:** Overview of the evidence tracks available in the UniTato v1.0 web server.

	Track name	Track description
**Gene models and gene model subsets**	UniTato-v4v6	DM Phureja potato unified (merged) v4 and v6 gene models (GFF)
	DMv4 unmatching v6	DM Phureja potato v4 gene models not matching v6 gene models (GFF), added to UniTato GFF
	DMv4 low-confidence matches to v6	DM Phureja potato v4 gene models matching v6 gene models with low confidence (GFF), added to UniTato GFF
	DMv4 high-confidence matches to v6	DM Phureja potato v4 gene models matching v6 gene models with high confidence (GFF), included in the translation table
**Pan-transcriptome**	Pan-transcriptome Desiree/PW363/Rywal	Representative *de-novo* assembled transcripts of potato cv. Desiree, cv. Rywal, and breeding clone PW363 (BAM)
**Long-read transcriptomes**	Rywal Iso-Seq transcripts	cv. Rywal potato Iso-Seq reads (BAM)
	Altus Iso-Seq transcripts	cv. Altus potato Iso-Seq reads (BAM)
	Avenger Iso-Seq transcripts	cv. Avenger potato Iso-Seq reads (BAM)
	Colomba Iso-Seq transcripts	cv. Colomba potato Iso-Seq reads (BAM)
	Spunta Iso-Seq transcripts	cv. Spunta potato Iso-Seq reads (BAM)
	PRJNA612026 ONT transcripts	Potato ONT reads from SRA project PRJNA612026 (BAM)
**Short paired-end read transcriptomes**	Phureja tuber Illumina PE	*Solanum tuberosum* L. Phureja Illumina NovaSeq 6000 reads (bw)
	Potato seed-tubers Illumina PE	*Solanum tuberosum* tuber-seeds from northern Antioquia/Cundinamarca/Boyaca (bw)
	Phureja DM1–3 516 R44 Illumina PE	*Solanum tuberosum* strain: DM1–3 516 R44 genome sequencing and assembly (bw)
	Phureja pistil Illumina PE	C065 pistil transcriptome sequencing (bw)
	Phureja Illumina PE	*Solanum phureja* lines contrasting by resistance to nematode (bw)
	Potato landraces young leaves Illumina PE	Transcriptomes of *in-vitro* young leaves in 11 potato landraces (bw)
	Phureja seed-tuber sprouts Illumina PE	RNA-Seq of certified and informal potato seed tubers in the province of Antioquia (bw)
**Reference proteomes**	Arabidopsis proteome Araport11	*Arabidopsis thaliana* proteome (v. Araport11) aligned to UniTato genome using miniprot
	Tomato proteome ITAG4.1	*Solanum lycopersicum* proteome (v. ITAG 4.1) aligned to UniTato genome using miniprot
	Tobacco proteome NtaSR1	*Nicotiana tabacum* proteome (v. SR1) aligned to UniTato genome using miniprot
	Benthi proteome NbeHZ1	*Nicotiana benthamiana* proteome (v. HZ1) aligned to UniTato genome using miniprot

### 2.3 UniTato improves the coverage and accuracy of gene models

Merging of v4 and v6 genome annotations improves the coverage and accuracy of the computationally predicted gene models (**Supplementary Table S3**), whereas manual annotation by experts will provide the necessary quality control. The improved coverage is most evident by adding the rescued v4 genes showing experimental evidence for expression. These include important genes, such as a gene encoding a cysteine protease inhibitor (PGSC0003DMG400010139/Sotub03g015980) and the salicylic acid-binding protein 2 (PGSC0003DMG400028777/Sotub06g025780; for details see Phureja_v4-v6.1_translations.xlsx on GitHub). Apart from the missing genes, several v6 genome models have been wrongly predicted. One such case is the TGA2 transcription factor gene encoded by two v6 gene models and correctly annotated as a single gene model by ITAG v4 (Tomaž et al., 2023). The Iso-Seq read mapping suggests that the gene’s 5′-untranslated region extends into another exon (**Figure 3**). Such mis-annotations can be easily manually curated in the UniTato Apollo instance. Here, tracks of mapped transcripts can additionally help curators build more accurate gene models (see **Supplementary Tables S3–S5**).

**Figure 4:**
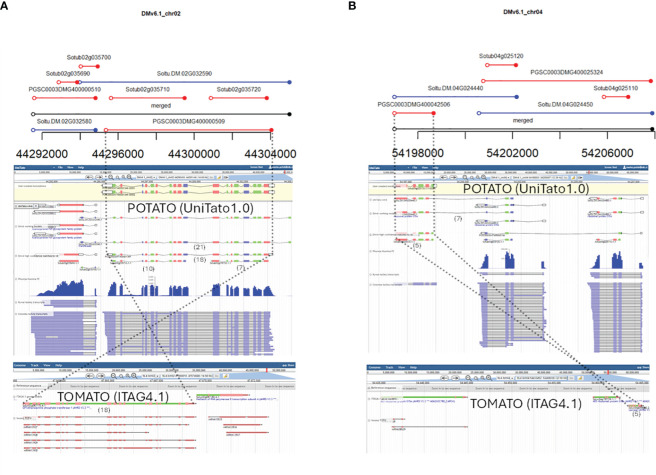
Examples of overlapping v4 and v6 gene models that require RNA-Seq read mapping and ortholog evidence for manual curation. From top to bottom: graphical representation of v4 and v6 gene model overlaps from “04_intervals_many-to-many.html” file on the UniTato GitHub repository, UniTato Apollo representation of these gene models with RNA-Seq Illumina PE and Iso-Seq tracks, and representation of tomato synthetic genomic region from the SolGenomics genome browser showing tomato ITAG4.1 annotation and Iso-Seq tracks. The numbers in brackets below the gene models show the exon count. Dotted lines follow the curated gene models through the three representations. **(A)** Manual curation of a v6 transferase gene model Soltu.DM.02G032590 for which the v4 model PGSCG0003DMT400001369 better fits the transcriptome data and the tomato ortholog evidence. **(B)** Manual curation of a chimeric v6 gene model Soltu.DM.04G024440 for which the v4 Sotub04g025130 model better fits the transcriptome and ortholog evidence.

We further decided to identify genomic loci where the v4 and v6 gene models were predicted very differently and/or overlap in a “many-to-many” fashion. Thus, without additional evidence, for these loci, it is very challenging to decide which gene models are more probable. A full list of such complex cases of gene models is available in “overlaps.xlsx” on the UniTato GitHub repository (**Supplementary Figure S2**). We showcase here two such genomic loci. The first is the v6 model Soltu.DM.02G032590 on chromosome 2 encoding a transferase gene (**Figure 4A**). The mapped Iso-Seq reads and the tomato ortholog sequence architecture with 18 exons fit better the v4 model PGSCG0003DMT400001369 than the v6 gene model. The second is the v6 gene model Soltu.DM.04G024440, a chimeric model of the laccase gene and the adjacent ribosomal protein S15A gene (**Figure 4B**). Based on the Iso-Seq data and the presence of only five exons in the tomato laccase ortholog, the v4 Sotub04g025130 gene model is more accurate.

## 3 Discussion

The advancement and maturation of high-throughput and long-read sequencing has led to several different potato genome assemblies, gene annotations, and transcriptomic datasets. Sequencing the group Phureja DM (Potato Genome Sequencing Consortium et al., 2011) still enables functional studies of polyploid potato cultivars using RNA-Seq technologies, although with the limitation of not covering cultivar-specific gene expression (Petek et al., 2020b; Hoopes et al., 2022). For practical reasons, most potato researchers use only one genome annotation, either PGSC (Potato Genome Sequencing Consortium et al., 2011) or ITAG (Tomato Genome Consortium, 2012), especially when conducting high-throughput analyses. However, using an incomplete gene set can lead to false outcomes regarding gene presence or gene family diversity, severely affecting downstream results (Yandell and Ence, 2012; Petek et al., 2020b). It is well known that incorrect or incomplete annotations corrupt all subsequent experiments that rely on them, making it essential to have the ability to share accurate and up-to-date annotations (Yandell and Ence, 2012; Bolger et al., 2018).

Our motivation here was thus two-fold: first, to transfer both gene model sets from the older PGSC assembly (Potato Genome Sequencing Consortium et al., 2011; Tomato Genome Consortium, 2012) to the new DMv6.1 assembly (Pham et al., 2020) and, at the same time, to merge the gene models (**Figure 2**), allowing for data interoperability of previous experimental results (e.g., from RNA-Seq) (Petek et al., 2020b) with the unified gene model set, UniTato. Annotation merging was performed using an in-house-developed bioinformatics pipeline that utilizes open-source software and complementing it with evidence from published tetraploid transcriptomes (Petek et al., 2020b) (**Figure 1**). The resulting annotation files were incorporated into an Apollo web server (Dunn et al., 2019), which enables the potato community to curate and refine potato gene models collaboratively and in real time, facilitating the establishment of a single standardized potato genome annotation (**Figure 3**). Moreover, by comparing v4 and v6 annotations with UniTato, we observed multiple complex cases of gene models that cannot be straightforwardly resolved and will need to be manually curated (see “overlaps.xlsx” on the UniTato GitHub for a list of gene identifiers for these complicated cases). We thus show how UniTato can be used to identify gene models that are either missing or were moved, merged, or split (see **Figures 3**, **4**; **Supplementary Figure S2**).

This showcases the usefulness of the established resource for resolving genome assembly and annotation issues. Bioinformatics users can thus i) compare gene models visually across versions and tracks, pinpointing and resolving errors and ensuring that the most accurate gene models are constructed and applied; ii) compare experimental results obtained on v4 to those obtained on the new v6 assembly or higher, such as for instance with RNA-Seq, where results with old identifiers can be incorporated with new results using v6 identifiers (via the translation table), without requiring repeated read mapping and computations; iii) curate potato gene models in problematic regions, such as determining gene structures in tandemly repeated gene regions, which cause problems with most annotation pipelines (multiple long-read and short-read tracks available in UniTato, see **Table 3**); and iv) use current data with popular genome analysis resources that still rely on older annotations (Van Bel et al., 2022; Yates et al., 2022), facilitating, e.g., translation of gene descriptions and ontologies via orthology from model plants. Furthermore, with the provided v4–v6 mapping and available evidence tracks (**Table 3**), UniTato also aids wet lab research. This includes i) guiding experiment design and interpretation, enabling users to check for off-target effects across different gene models; ii) defining and cloning functional orthologs based on experimental results and not merely partial sequence similarity, by revealing if orthologs from another plant map to the v6 assembly (see RNA-Seq and proteome tracks, **Table 3**); and iii) primer design, since the unified gene models are an improvement over the initial v4 and v6 models, enhancing gene coverage and accuracy (e.g., the user can visually determine if the amplicon is covering variations in the RNA-Seq tracks, **Table 3**).

In conclusion, we believe that building upon existing gene models to improve and unify them in a community-wise manner is a reasonable and transparent way to improve potato gene model annotations. The repeated creation of new genome model versions, without interlinking, is not contributing to the FAIR data paradigm (Wilkinson et al., 2016; Petek et al., 2022) and thus hinders agricultural research, including precision agriculture and food safety (Cole et al., 2018). The requirements of periodic annotation curation and incorporating experimental data and novel findings into the annotation process are inherent also to other plant species (Yandell and Ence, 2012; Kersey, 2019). Even in model plants, up to 40% of protein-coding genes can still be of unknown function, suggesting that much work is still required to fully resolve, annotate, and understand most plant genomes (Horan et al., 2008; Wang et al., 2023). We propose that a similar approach for evidence- and community-based revision as the one presented here can be utilized for any other insufficiently annotated species, for which genome models of closely related species are available. Apart from updating our database with new assemblies as they become available (Yang et al., 2023), future developments include the addition of novel experimental omics datasets and expansion to related genomes.

## 4 Methods

### 4.1 Data sources

To develop UniTato, we used the publicly available potato group Phureja DM gene models: DMv6.1 (Pham et al., 2020), ITAG (Tomato Genome Consortium, 2012), and PGSCv4.04 (Potato Genome Sequencing Consortium et al., 2011), as well as reference transcriptomes of Désirée, Rywal, and PW363 tetraploid genotypes, and an ITAG/PGSC translation table (Petek et al., 2020b). The latter consolidated the two publicly available PGSC and ITAG gene models into a single unified one.

### 4.2 Data processing

To map gene annotations across potato genome assemblies (**Figure 1**), GFF files were sorted using the *sort* function from Bedtools v2.25.0 (Quinlan and Hall, 2010). Liftoff v.1.6.3 (Shumate and Salzberg, 2020) uses Minimap2 (Li, 2018) to map annotations between assemblies of the same or closely related species. We modified it to accept the number of nucleotides for the *flank* parameter (https://github.com/NIB-SI/Liftoff), instead of the ratio of sequence size, and used with the following parameters: i) coverage of 0.90%, ii) sequence identity of 90%, iii) flanking sequence length *flank* of either 0 or 500 nucleotides, and iv) Minimap2 v.2.24-r1122 “asm5” option for long assembly to reference mapping. In addition, Minimap2 was used with the same set of parameters as for Liftoff (–end-bonus 5 –eqx -N 50 -p 0.8 -ax asm5) to map the reference CDSome and transcriptome (Petek et al., 2020b) of three potato genotypes: Désirée, PW363, and Rywal. FASTQ files of long-read transcriptomics datasets were downloaded from SRA. The Iso-Seq reads were mapped to the v6 genome assembly using Minimap2 with parameters “-ax splice:hq -G 10000 -uf”.

Next, to compare the mapped annotation across the assemblies, as well as overlaps within the DMv6.1, the *intersect* function from Bedtools (Quinlan and Hall, 2010) was used with the following minimum overlap as a fraction (*F*) ranging incrementally from 0.0001 to 1. Pairs from our existing merged v4 genome model (Petek et al., 2020b) were used to determine the optimal *F* threshold value of 0.30 (**Supplementary Figure S1**). All reported v6 values below refer to working model versions unless stated otherwise. To obtain additional evidence about the reliability of the transcriptome-unsupported rescued genes, we mapped i) short paired-end Illumina RNA-Seq reads of DM Phureja (Pham et al., 2020) using STAR (Dobin et al., 2013) and of cv. Rywal (Lukan et al., 2020) using Salmon (Patro et al., 2017); ii) long reads of tetraploid cultivars (Della Bartola et al., 2020; Lukan et al., 2020; Hoopes et al., 2022) using Minimap2 (Li, 2018); and iii) protein sequences of the model plant *Arabidopsis* (Cheng et al., 2017; Pasha et al., 2020) and three Solanaceae species (Hosmani et al., 2019; Wang et al., 2024) using miniprot v.0.13-r24 (-G 100 -O 10 -J 34 -F 30 -j1 -M0 –gff-only -ut64) (Li, 2023).

For visualization, packages circlize v0.4.15 (Gu et al., 2014) and intervals v0.15.4 (github.com/edzer/intervals) were used with default settings. For topological sorting of unified GFF features, AGAT v0.6.2 (Dainat et al., 2023) was used with default settings.

### 4.3 Database implementation

A web server hosting the Apollo genomic annotation editor (Dunn et al., 2019) for real-time collaborative analysis and curation was deployed at https://unitato.nib.si. The reference DM genome assembly (DMv6.1) was uploaded as the base organism. Several evidence tracks corresponding to the different gene models are available for exploration and curation. The database instance is running Apollo 2.7.0, deployed with docker, with default parameters. Data upload was carried out using JBrowse utility scripts (Buels et al., 2016).

### 4.4 Software and code

The programming environments R v.4.3 (https://www.r-project.org/) and Python v3.8 (https://www.python.org/) were used. Code to reproduce the analysis and results including scripts used for constructing the mapping table between v4 and v6 gene IDs, as well as merging v4 and v6 models are available at the GitHub repository (https://github.com/NIB-SI/unitato).

## Data availability statement

Data and code to reproduce the analysis are available at the GitHub repository https://github.com/NIB-SI/unitato/. The GFF and GTF files and the identifier translation table are also available from https://unitato.nib.si/downloads/. Publicly available RNA-Seq datasets were used in the study. The data can be found under the following SRA accession numbers: SRR8281993-SRR8282008 (Rywal IsoSeq reads; SRA study SRP172523), SRR14298411-SRR14298459 (Altus, Avenger, Colomba and Spunta IsoSeq reads; SRA study SRP315827), SRR11431596-SRR11431617 (PRJNA612026 ONT reads; SRA study SRP254248), SRR10690850, SRR10690852, SRR10690854, SRR10690856, SRR10690857, SRR10690858 (Rywal Illumina reads; SRA study SRP237525; GEO accession GSE142002), SRR122108, SRR122109, SRR122113, SRR122122, SRR122124, SRR122129, SRR122139 (Phureja Illumina reads; SRA study SRP005965), SRR7047512 (Phureja Illumina reads; SRA study SRP141363), SRR8457030-SRR8457059 (Phureja Illumina reads; SRA study SRP180310), SRR14627804-SRR14627805 (Phureja Illumina reads; SRA study SRP321011), SRR17202512-SRR17202515 (Phureja Illumina reads; SRA study SRP350333), SRR17244262-SRR17244298 (Phureja Illumina reads; SRA study SRP350981), and SRR10153126 (Phureja Illumina reads; SRA study SRP222783).

## Author contributions

MZ: Conceptualization, Data curation, Formal analysis, Investigation, Methodology, Software, Validation, Visualization, Writing – original draft, Writing – review & editing. JZ: Formal analysis, Funding acquisition, Investigation, Methodology, Writing – original draft, Writing – review & editing. CB: Formal analysis, Funding acquisition, Investigation, Methodology, Software, Visualization, Writing – review & editing. NN: Formal analysis, Investigation, Validation, Writing – review & editing. MJ: Formal analysis, Investigation, Validation, Writing - review and editing. ŽR: Investigation, Writing – review & editing. KG: Conceptualization, Investigation, Project administration, Supervision, Writing – review & editing, Funding acquisition, Resources. MP: Conceptualization, Data curation, Formal analysis, Funding acquisition, Investigation, Methodology, Project administration, Resources, Supervision, Validation, Visualization, Writing – original draft, Writing – review & editing.
