# Supplementary material for: Evidence-based unification of potato gene models with the UniTato collaborative genome browser
Source: Front Plant Sci. 2024 Jun 11;15:1352253. doi: 10.3389/fpls.2024.1352253 (PMC11196761; doi:10.3389/fpls.2024.1352253)
Supplement: Supplementary file 1 [file DataSheet_1.pdf]

Supplementary Material

Evidence based unification of potato gene models with UniTato collaborative genome browser

Maja Zagorščak[1†], Jan Zrimec[1†], Carissa Bleker[1], Nadja Nolte[1], Mojca Juteršek[1], Živa Ramšak[1], Kristina Gruden[1] & Marko Petek[1,*]

[1] National Institute of Biology, Večna pot 121, 1000 Ljubljana, Slovenia

[†] These authors contributed equally to this work and share first authorship

* corresponding author email: marko.petek@nib.si

Contents

Supplementary figures … p2 - p3
Supplementary tables … p4 - p8
Supplementary references … p9

Supplementary figures

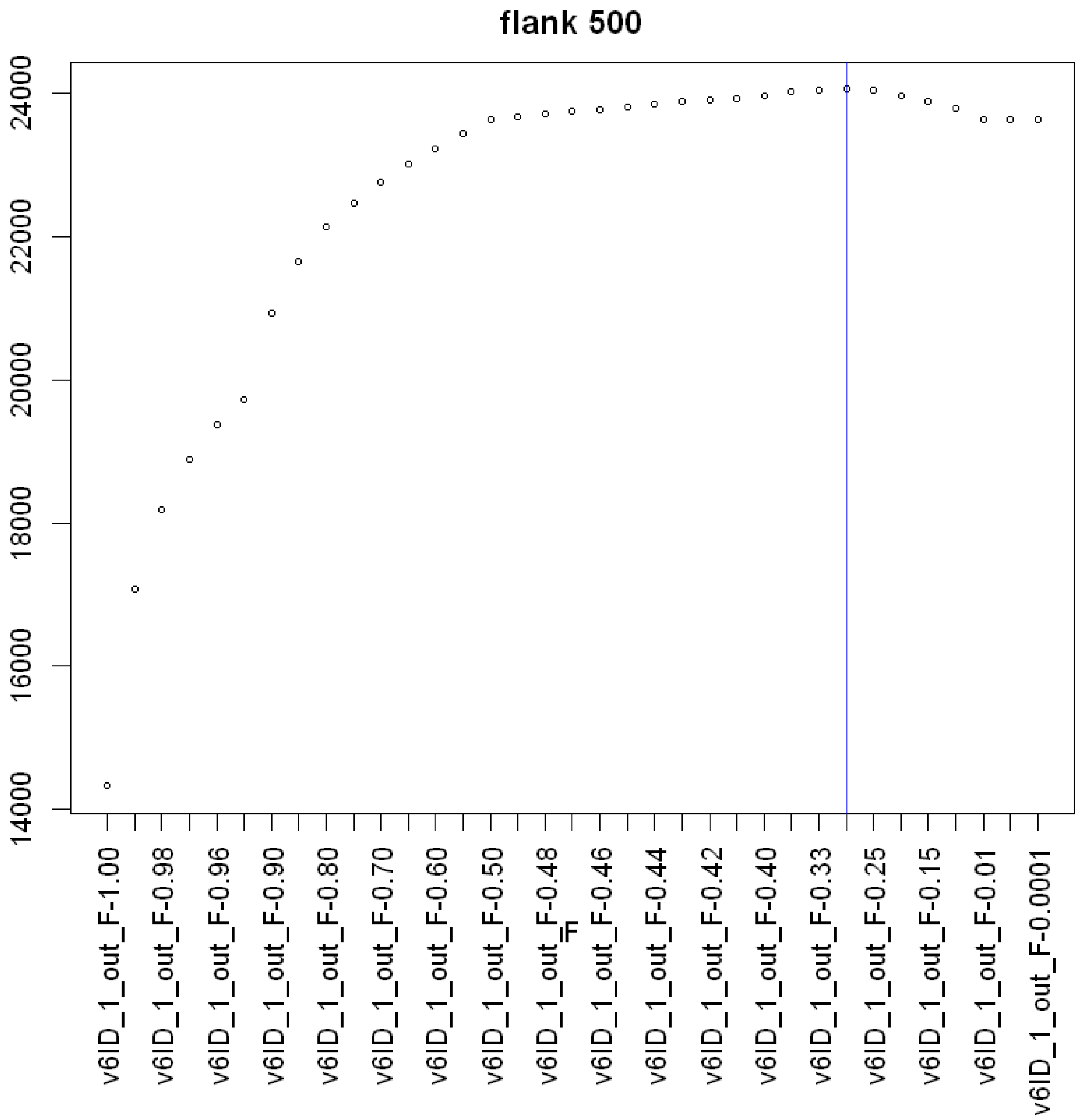

Figure S1. Bedtools optimal F threshold selection using ITAG/PGSC pairs mapped to v6 genome with Liftoff (with *flank* = 500) defined in the merged v4n gene model (Petek et al. 2020). The graph shows the number of v4 ITAG/PGSC pairs mapped to the same v6 gene model at decreasing Bedtools F values.

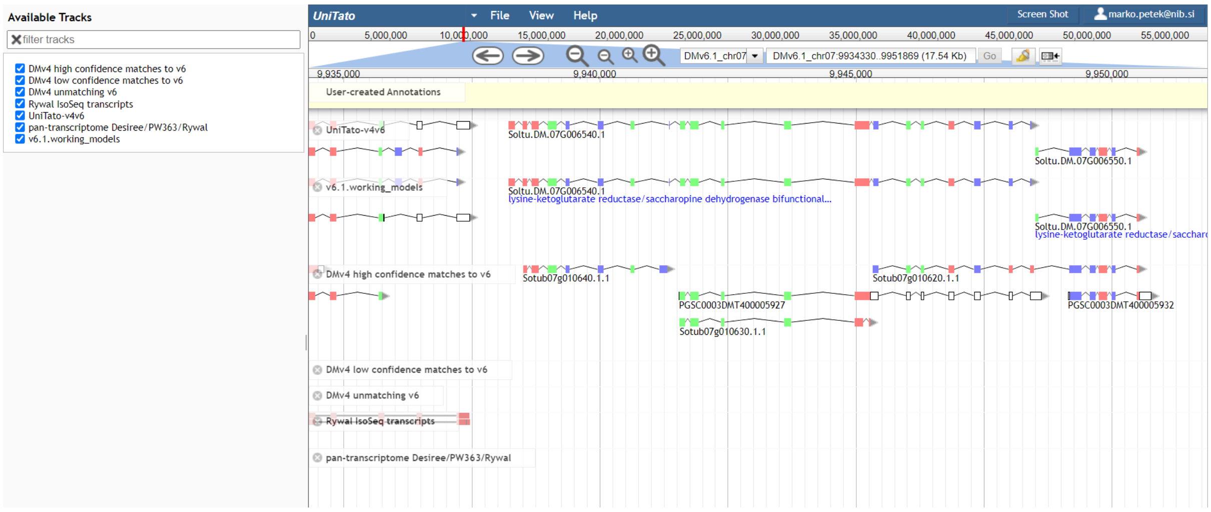

Figure S2. An example of v4 and v6 gene models that in our opinion cannot be simply resolved and requires manual curation, possibly based on more evidence. For a list of such gene models see overlaps.xlsx on the Unitato GitHub page (github.com/NIB-SI/unitato).

Supplementary tables

Table S1. Flank preference in UniTato-v4v6 GFF generation. Mapped genome models with Bedtools coverage F < 0.30 were added to the v6.1 working model GFF3, thus defining UniTato-v4v6. Note: in most cases, genome models from mappings with flank 500 nt were selected. Considering both mappings, without flank and using flank 500 nt, only 527 v4 genome models did not map to the v6, from which 316 were from the PGSC and 211 were from the ITAG annotation. DMv6 high-confidence genome models are defined in (Pham et al. 2020).

	**ITAG/PGSCv4**	**DMv6.1**	**DMv6.1 high-confidence**
**Total gene model count**	74,432	40,652	32,917
**F >= 0.30 flank 0**	387	458	368
**F >= 0.30 flank 500**	56,776	31,594	29,065
**0.0001 <= F < 0.30 flank 0**	13	19	14
**0.0001 <= F < 0.30 flank 500**	1,142	1,418	1,028
**0 <= F < 0.0001 flank 0**	32	/	/
**0 <= F < 0.0001 flank 500**	15,555	/	/
**unmapped**	527	/	/

Table S2. Summary of potato genomes and annotations used in the study.

Line / accession	Assembly version	Ploidy	Est. genome size	Annotation	Gene number	Gene model / transcript number
DM	v4.04	n	840 Mbp	ITAG v4 (Sotub)	35,004	35,004
DM	v4.04	n	840 Mbp	PGSC v4 (PGSCG)	39,431	39,431
DM	v6.1	n	740 Mbp	v6 (Soltu.)	40,652	52,953
Desiree	pan-transcriptome 2020	4n	NA	/	NA	57,943
Rywal	pan-transcriptome 2020	4n	NA	/	NA	43,883
PW363	pan-transcriptome 2020	4n	NA	/	NA	36,336
Altus	v1	4n	2140 Mbp	v1	108,303	113,629
Avenger	v1	4n	2346 Mbp	v1	113,401	119,820
Colomba	v1	4n	1990 Mbp	v1	100,057	106,091
Spunta	v1	4n	1992 Mbp	v1	98,572	103,511

Table S3. Number of UniTato genes covered by reads from at least one run from *Solanum phureja* RNA-Seq experiments. Illumina Paired-End reads mapped with STAR; various number of samples per experiment.

	**SRP005965**	**SRP141363**	**SRP180310**	**SRP222783**	**SRP321011**	**SRP350333**	**SRP350981**
**Num. DMv6.1 geneIDs**	31,192 (76.7%)	23,112 (56.9%)	33,542 (82.5%)	28,309 (69.6%)	31,582 (77.7%)	30,635 (75.4%)	30,779 (75.7%)
**Num. UniTato v4 rescued geneIDs**	4,495 (26.8%)	2,317 (13.8%)	5,677 (33.9%)	3,315 (19.8%)	3,223 (19.3%)	4,395 (26.3%)	4,828 (28.8%)

Table S4. Comparisons of mapping RNA-Seq reads from tetraploid potato cultivar Rywal to UniTato or DMv6.1. Six RNA-Seq samples (three potato virus Y infected - Inf, and three control - Ctrl samples of cv. Rywal) were mapped with Salmon (Patro et al. 2017) to DMv6.1 genome reference (Pham et al. 2020) using either DMv6.1 or UniTato genome annotation files. Reads - total number of raw reads in each sample. ReadCount_v6 and readCount_UniTato - read count over all genes in Salmon output was summed for each sample to obtain information on mapping and quantification with either of the two annotation files. ReadCount_UniTato_v4 - number (and percent) of counts assigned to PGSC/ITAGv4 gene models within the UniTato annotation. Analysed samples were part of a larger published study (Lukan et al. 2020) and are deposited in SRA under given accession numbers.

Sample	Reads	readCount_v6	readCount_UniTato	readCount_UniTato_v4
Inf_1<br>SRR10690850	26,190,584	20,349,876	20,635,229	403,246 (1.95 %)
Inf_2<br>SRR10690852	23,837,179	17,588,269	17,950,810	455,404 (2.54 %)
Inf_3<br>SRR10690854	21,825,967	16,238,976	16,620,190	467,864 (2.82 %)
Ctrl_1<br>SRR10690856	26,176,181	19,237,809	19,708,517	530,784 (2.69 %)
Ctrl_2<br>SRR10690857	22,659,230	16,010,890	16,624,179	665,217 (4.00 %)
Ctrl_3<br>SRR10690858	20,311,766	15,115,518	15,377,755	306,268 (1.99 %)

Table S5. Comparing expression of genes annotated with PGSC/ITAGv4 or DMv6.1 gene models when mapping RNA-Seq reads from tetraploid potato cv. Rywal to UniTato. In the first column are numbers of UniTato gene models originating from either PGSCv4, ITAGv4, or DMv6.1 annotation together with percents out of all UniTato gene models. In the second column are numbers of expressed genes within each gene model group, obtained by filtering low expressed genes using edgeR. Percents in brackets represent the portions of expressed genes within each gene model group. In the third column are numbers of differentially expressed genes (pAdj < 0.05, |logFC| > 1) within each gene model group, obtained after limmaVoom differential expression analysis. Percents in brackets represent the portions of differentially expressed genes within expressed genes of each gene model group.

**Gene model groups**	**Salmon output**<br>Number (percent of all UniTato gene models)	**Expressed**<br>Number (percent per gene model group)	**Differentially expressed**<br>Number (percent per expressed gene models in each group)
DMv6.1	52,940 (76.0 %)	19,583 (37.0 %)	7,222 (36.9 %)
PGSCv4	11,513 (16.5 %)	471 (4.1 %)	151 (32.1 %)
ITAGv4	5,229 (7.5 %)	159 (3.0 %)	46 (28.9 %)
UniTato	69,682 (100.0 %)	20,213 (29.0 %)	7,419 (36.7 %)

Supplementary references

Lukan, Tjaša, Maruša Pompe-Novak, Špela Baebler, Magda Tušek-Žnidarič, Aleš Kladnik, Maja Križnik, Andrej Blejec, et al. 2020. “Precision Transcriptomics of Viral Foci Reveals the Spatial Regulation of Immune-Signaling Genes and Identifies RBOHD as an Important Player in the Incompatible Interaction between Potato Virus Y and Potato.” *The Plant Journal: For Cell and Molecular Biology* 104 (3): 645–61.

Patro, Rob, Geet Duggal, Michael I. Love, Rafael A. Irizarry, and Carl Kingsford. 2017. “Salmon Provides Fast and Bias-Aware Quantification of Transcript Expression.” *Nature Methods* 14 (4): 417–19.

Petek, Marko, Maja Zagorščak, Živa Ramšak, Sheri Sanders, Špela Tomaž, Elizabeth Tseng, Mohamed Zouine, Anna Coll, and Kristina Gruden. 2020. “Cultivar-Specific Transcriptome and Pan-Transcriptome Reconstruction of Tetraploid Potato.” *Scientific Data* 7 (1): 249.

Pham, Gina M., John P. Hamilton, Joshua C. Wood, Joseph T. Burke, Hainan Zhao, Brieanne Vaillancourt, Shujun Ou, Jiming Jiang, and C. Robin Buell. 2020. “Construction of a Chromosome-Scale Long-Read Reference Genome Assembly for Potato.” *GigaScience* 9 (9). https://doi.org/10.1093/gigascience/giaa100.
